# TFRC in cardiomyocytes promotes macrophage infiltration and activation during the process of heart failure through regulating Ccl2 expression mediated by hypoxia inducible factor‐1α

**DOI:** 10.1002/iid3.835

**Published:** 2023-08-28

**Authors:** Yanyun Pan, Jinxiu Yang, Jin Dai, Xiaoming Xu, Xinbin Zhou, Wei Mao

**Affiliations:** ^1^ Department of Cardiology The First Affiliated Hospital of Zhejiang Chinese Medical University Hangzhou Zhejiang Province P. R. China; ^2^ Department of Cardiology, The First Affiliated Hospital, School of Medicine Zhejiang University Hangzhou Zhejiang Province P. R. China

**Keywords:** cardiac hypertrophy, C‐C motif ligand 2, hypoxia‐inducible factor‐1α, signal transducer and activator of transcription 3, transferrin receptor

## Abstract

**Background:**

Cardiac hypertrophy is an initiating link to Heart failure (HF) which still seriously endangers human health. Transferrin receptor (TFRC), which promotes iron uptake through the transferrin cycle, is essential for cardiac function. However, whether TFRC is involved in the process of pathological cardiac hypertrophy is not clear.

**Methods:**

Transverse aortic constriction (TAC) mouse model and mice primary cardiomyocytes treated with isoproterenol (ISO) or phenylephrine (PHE) were used to mimic cardiac hypertrophy in vivo and in vitro. Single cell RNA sequence data from heart tissues of TAC‐model mice was obtained from the Gene Expression Omnibus (GEO) database, and was analyzed with R package Seurat. TFRC expression and macrophage infiltration in the heart tissue were tested by immunofluorescent staining. Macrophage polarization was detected by Flow Cytometry. TFRC expressions were detected by qRT‐PCR, Western blot, and ELISA.

**Results:**

TFRC expression is increased in the pathological cardiac hypertrophy of mice model and positively associated with macrophage infiltration. Furthermore, TFRC in cardiomyocytes recruits and activates macrophages by secreting C‐C motif ligand 2 (Ccl2) in the mice heart tissue with TAC surgery or in the primary cardiomyocytes stimulated with ISO or PHE to induce myocardial hypertrophy in vitro. Moreover, we find that TFRC promotes Ccl2 expression in cardiomyocytes via regulating signal transducer and activator of transcription 3 (STAT3). In addition, we find that increased TFRC expression in the HF tissue is regulated by hypoxia‐inducible factor‐1α (HIF‐1α).

**Conclusion:**

This in‐depth study shows that TFRC in cardiomyocytes promotes HF development through inducing macrophage infiltration and activation via the STAT3‐Ccl2 signaling, and TFRC expression in cardiomyocytes is regulated by HIF‐1α during HF. This study first uncovers the role of TFRC in cardiomyocytes on macrophage infiltration and activation during HF.

## Graphical abstract

TFRC in cardiomyocytes promoted heart failure (HF) development through inducing macrophage infiltration and activation via the STAT3‐Ccl2 signaling, and TFRC expression in cardiomyocytes was regulated by HIF‐1α during HF.

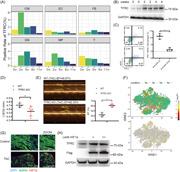

## 1 INTRODUCTION

Heart failure (HF), as a global pandemic with high incidence and prevalence, still seriously endangers human health.^1^ Generally, the morbidity of HF is 1–20 cases per 1000 people in adult.^2^ Besides, among HF patients, 51% of patients have severe HF.^3^ Nowadays, several risk factors for HF have been identified, including smoking, arterial hypertension, diabetes mellitus, excess body weight, male gender, age, and cardiac hypertrophy which is considered as the initiating link to HF.^4^, ^5^ Thus, preventing pathological cardiac hypertrophy is a potential strategy for treating HF.

Iron, which is important for oxidative phosphorylation and oxygen transport, plays essential roles in cardiomyocyte maintenance and function.^6^ Both iron overload and insufficiency could lead to HF.^7^, ^8^ Transferrin receptor (TFRC) promotes iron uptake through the transferrin cycle by facilitating receptor mediated endocytosis of iron.^9^ TFRC specific knockout in the heart leads to death in the second week of mice life with cardiomegaly and poor cardiac function.^10^ However, whether TFRC is involved in the process of pathological cardiac hypertrophy is not clear.

Besides cardiomyocytes, noncardiomyocytes, including macrophages, fibroblasts, and so on, play important roles in pathological cardiac hypertrophy.^11^, ^12^ Interestingly, immune checkpoint inhibitor therapy‐related cardiotoxicity and heart failure are related to immune‐infiltration of T lymphocytes, macrophages and neutrophils in myocardial tissues which is probably associated with activation of NLRP3‐MyD88‐chemokine pathways and increased expressions of systemic SDF‐1, cardiac DAMPs Fibronectin‐EDA, S100/Calgranulin, and galectine‐3 in cardiac tissues.^13^, ^14^, ^15^, ^16^ Heart‐resident macrophages are activated into pro‐inflammatory M1 type to trigger inflammatory response, resulting in accelerated deterioration of cardiac function in HF.^11^, ^12^, ^17^ Cardiac fibroblasts are activated to trigger myocardial fibrosis in HF.^18^, ^19^ Single‐cell RNA sequencing of pressure overload‐induced pathological cardiac hypertrophy in mice showed that macrophage activation and subtype switching, a key event at middle‐stage of cardiac hypertrophy.^20^ Targeting macrophages in hypertrophy during the switch from normal to reduced cardiac function may attenuate disease progression.^20^ Recently, TFRC is identified as a prognostic biomarker which correlates with immune infiltration in breast cancer.^21^ In addition, TFRC expression in induced sputum is positively correlated with the number of pro‐inflammatory M1 macrophages and asthma severity.^22^ Whether TFRC in the heart is associated with macrophage activation in HF needs to be explored.

In this study, we examined the changes of TFRC expression in the heart tissues during the process of cardiac hypertrophy using the transverse aortic constriction (TAC) mouse model and the primary cardiomyocytes stimulated with isoproterenol (ISO) or phenylephrine (PHE) to induce myocardial hypertrophy in vitro, and explored the potential role of TFRC on myocardial hypertrophy.

## 2 MATERIALS AND METHODS

### 2.1 Single‐cell RNA sequencing (scRNA‐seq) analysis

scRNA‐seq data from heart tissues of TAC‐model mice was obtained from the Gene Expression Omnibus (GEO) database (GSE120064), and was analyzed with R package Seurat as previously described.^19^, ^23^

### 2.2 Transverse aortic constriction (TAC) mouse model

This study was approved by the institutional animal care committee of the First Affiliated Hospital of Zhejiang Chinese Medical University and complied with the Guide for the Care and Use of Laboratory Animals. Eight‐week‐old male C57BL/6J mice were purchased from Charles River. Animals were housed at room with a temperature of 18–23°C and 50%–60% humidity, with a 12/12 h light/dark cycle. All the animals were provided with standard food and tap water. HF was induced in mice by TAC, as previously described.^24^, ^25^ Breiefly, the mice were anesthetized, fixed on the transparent board in a supine position and connected to a small animal ventilator through laryngotracheal intubation. The aortic arch was exposed, and the transverse aortic arch was tied with a 7‐0 silk suture between the brachiocephalic and left common carotid arteries using an overlaying 27G needle. The constriction needle was carefully removed, the chest and skin incision wounds were closed using a 4‐0 silk suture, and the ventilator was disconnected. Mice were placed on warm pads until they woke up after surgery. The sham group underwent the same surgical procedure without aortic banding. Moreover, to knockdown of TFRC or Ccl2, AAV9 viral delivery of control shRNA (AAV9‐shR‐Ctl), shRNA against TRFC (AAV9‐shR‐TRFC), and AAV9‐shR‐Ccl2 were obtained from Genepharma. Mice were injected with AAV9 (4 × 10^11^ vector genomes (vg)/mouse) via the tail vein. For experiments, mice were randomly divided into Sham group (as control) (*n* = 6), TAC group (*n* = 6), Sham+AAV9‐shR‐Ctl group (*n* = 6), Sham+AAV9‐shR‐TRFC group (*n* = 6), TAC+AAV9‐shR‐Ctl group (n = 12), TAC+AAV9‐shR‐TRFC group (*n* = 6), and TAC+AAV9‐shR‐Ccl2 group (*n* = 6), TAC+macrophages infected with Lenti‐shR‐Ctl group (*n* = 6), and TAC+macrophages infected with Lenti‐shR‐TRFC group (*n* = 6), respectively.

### 2.3 Immunofluorescent staining

Cardiac tissues were fixed in 4% paraformaldehyde, cut into slices, and embedded with paraffin. After dewaxing, slides were batched for 20 min at 80°C, and then incubated with TRFC antibody (ab214039; Abcam), α‐actinin antibody (ab90421; Abcam), F4/80 antibody (aab6640; Abcam), Hif‐1α antibody (ab216842; Abcam), at 4°C overnight, and treated with fluorescence‐conjugated secondary antibody for 1 h at 37°C. The nuclei were stained with DAPI. After washing, the sections were imaged by a fluorescence microscope.

### 2.4 LV end‐diastolic diameter (LVEDd) and ejection fraction [EF (%)] measurement

LVEDd and EF (%) are measured, as previously described.^26^ Briefly, 4 weeks after TAC surgery, the cardiac function of mice by echocardiography using a 30‐MHz high‐frequency scanhead (VisualSonics Vevo770; VisualSonics). End‐systole and end‐diastole were defined as the phases in which the smallest and largest areas of the left ventricular (LV), respectively, were obtained. LVEDd were measured from the LV M‐mode at the mid‐papillary muscle level. Image frames were acquired in the parasternal long axis and the parasternal short axis. Left ventricular ejection fraction was determined using the tool “LV Trace” from Vevo LAB software.

### 2.5 Cell culture

Mouse cardiomyocyte isolation was performed as published.^23^, ^27^ Raw264.7 macrophages were stored in our lab, and cultured at 37°C under 5% CO_2_ humidified atmosphere, with DMEM supplemented with FBS (10%), Penicillin (100 U/mL) and Streptomycin (100 μg/mL). For inducing hypertrophy, cardiomyocytes were treated with treated with isoproterenol (ISO, 10 μmol/L, Sigma) or phenylephrine (PHE, 50 μmol/L, Sigma) for 24 h. To explore the effect of STAT3 on Ccl2 expresson in cardiomyocytes, cardiomyocytes were pretreated with the STAT3 inhibitor, Stattic (5 μM, Sigma) for 2 h, and then stimulated with 50 μm FeSO_4_ (Sigma) for 4 h. Then, the cells were collected for further examination. To knockdown of TFRC or overexpression of HIF‐1α, the macrophages isolated from the mice heart tissue were infected with Lentivirus‐shR‐TFRC, Lenti‐flag‐HIF‐1α, or Lenti‐control obtained from Genepharma for 48 h.

### 2.6 Quantitative real‐time PCR (qRT‐PCR)

qRT‐PCR was performed as previously published.^28^ Primers were as follows: chemokine (C‐C motif) ligand 2 (Ccl2), forward 5ʹ‐TTAAAAACCTGGATCGGAACCAA‐3ʹ, reverse 5ʹ‐GCATTAGCTTCAGATTTACGGGT‐3ʹ; GAPDH, forward 5ʹ‐TGGCCTTCCGTGTTCCTAC‐3ʹ, reverse 5ʹ‐GAGTTGCTGTTGAAGTCGCA‐3ʹ. Relative gene expression was obtained by the ΔΔ*C*_T_ methods, and the relative gene expression levels were expressed in terms of fold induction ($2^{‐\Delta \Delta C_{T}}$).

### 2.7 Enzyme‐linked immunosorbent assay (ELISA)

Ccl2 protein expression in cell culture medium of mouse cardiomyocytes or the serum were determined by the Mouse MCP1 ELISA Kit (ab100722; Abcam), according to the manufacturer's protocol.

### 2.8 Flow cytometry

To evaluate the effects of TRFC expression in mouse cardiomyocytes on macrophages, RAW264.7 macrophages seeded in the 6‐well plate were co‐cultured with mouse cardiomyocytes which isolated from the heart tissue of mice with TAC surgery and infected with AAV9‐shR‐Ctl or AAV‐9‐shR‐TRFC and seeded in the upper compartment of a transwell chamber apparatus. After 24 h, RAW264.7 macrophages were collected, fixed and permeabilized using a BD fix/perm kit. Then, cells were stained with a PE‐conjugated anti‐mouse IFN‐gamma antibody (TONBO; 50‐7311‐U025), and APC‐conjugated anti‐mouse iNOS antibody (theromofisher; 17‐5920‐82) and then analyzed with Flow Cytometry, as previously described.^29^ To evaluate the effects of Ccl2 expression on macrophages in the heart tissue, macrophages from the heart tissue of mice with TAC surgery and infected with AAV9‐shR‐Ctl or AAV‐9‐shR‐Ccl2 were isolated using magnetic microbeads as previously described,^30^, ^31^ and then analyzed with Flow Cytometry.

### 2.9 Western blotting

Western blotting was performed as previously described.^28^ Antibodies against STAT3 (phospho S727) (pSTAT3, ab32143; Abcam), STAT3 antibody (ab119352; Abcam), GAPDH (ab181602; Abcam), Flag (ab205606; Abcam), TFRC (ab214039; Abcam) were used.

### 2.10 Statistical analysis

Statistical analysis was assessed using SPSS 16.0. All data are presented as mean ± SD. Values of *p* < .05 were considered significant. For the comparison of two paired samples with normal distribution, a paired *t*‐test was used. When symmetrically distributed, a Wilcoxon test was used. For the comparison of two unpaired samples with normal distribution plus similar variances, an unpaired *t*‐test was used. When variances differed significantly, Welch's *t*‐test was used. When there was no normal distribution, a Mann–Whitney test was used. For multiple comparisons with normal distribution, the Ordinary One‐Way ANOVA with Holm‐Sidak's multiple comparisons test/Tukey's multiple comparisons test as post hoc test was used. For multiple comparisons without normal distribution, a Kruskal–Wallis‐test with Dunn's multiple comparisons test as post hoc‐test was used.

## 3 RESULTS

### 3.1 TFRC expression is increased in the heart tissue with pathological cardiac hypertrophy of mice model and positively associated with macrophage infiltration

**Figure 1:**
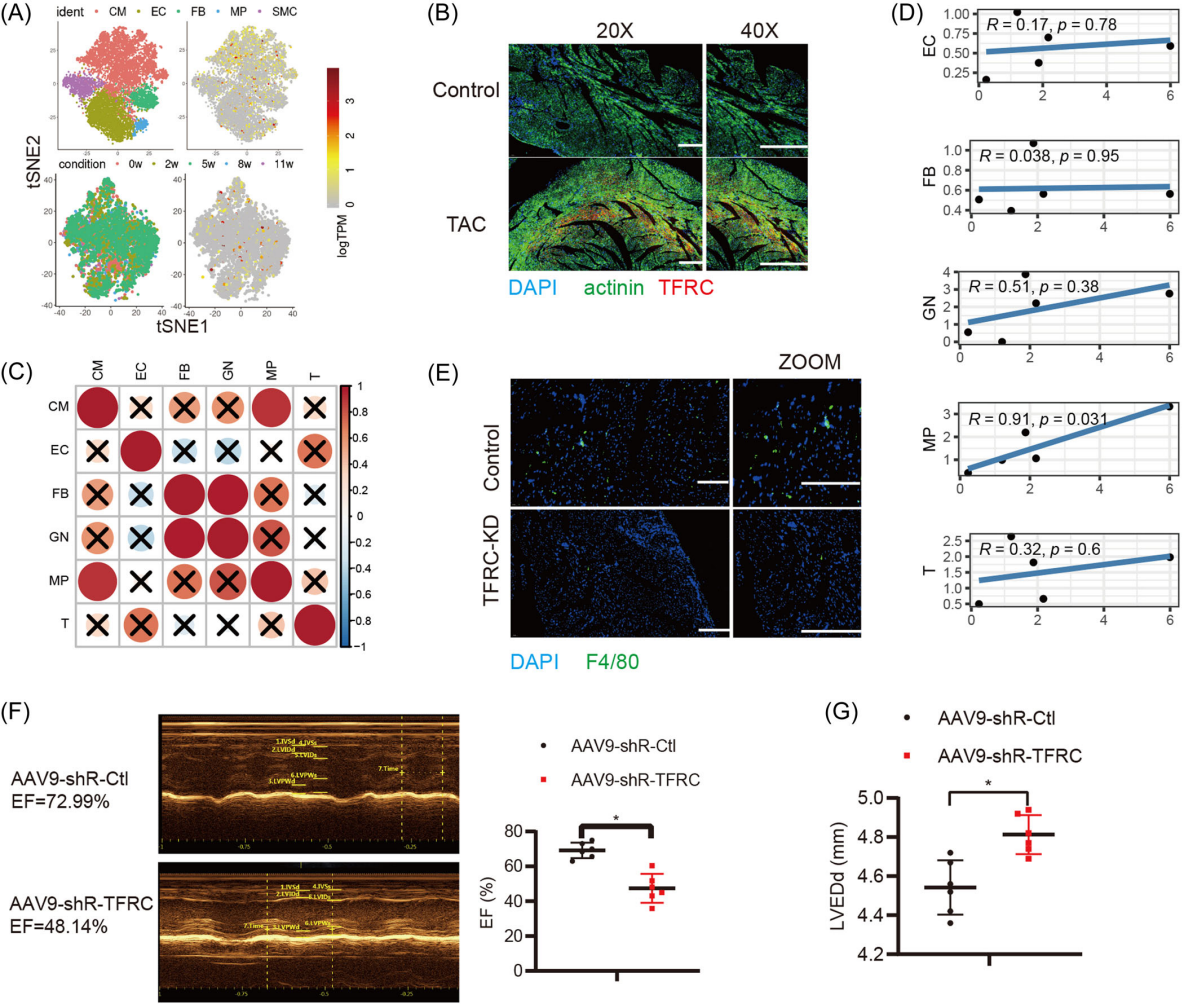
TFRC expression is increased in the heart tissue with pathological cardiac hypertrophy of mice model and positively associated with macrophage infiltration. (A) 2D visualization of TRFC gene expressions in single‐cell clusters of TAC‐model mouse heart tissues by tSNE. CM, cardiomyocytes; EC, endothelial cells; FB, fibroblasts; MP, macrophages; SMC, smooth muscle cells) (upper); 2D visualization of TRFC gene expressions at different stages of cardiac hypertrophy (down). (B) Immunofluorescence analysis of TRFC (Red) expressions in the heart tissue of mice subjected to TAC for 4 weeks. The cardiomyocytes were stained with anti‐α‐actinin antibodies (Green). Nucleus was stained with DAPI (Blue). The bar is 50 μm. (C) The correlation analysis of different cells in HF tissue. (D) The correlation analysis of TRFC expression and different cells in HF tissue (CM, cardiomyocytes; EC, endothelial cells; FB, fibroblasts; MP, macrophages; GN, neutrophile granulocyte; T, T cells). (E) Immunofluorescence analysis of macrophage distribution stained with anti‐F4/80+ antibodies (Green) in HF tissue of mice injected with AAV9‐shR‐Ctl or AAV‐9‐shR‐TFRC through tail vein for 3 weeks, then subjected to TAC for 4 weeks. The bar is 100 μm. (F) Representative images of echocardiography and quantification of ejection fraction (EF) % (*n* = 6). (G) Detection of left ventricular end‐diastolic diameter (LVEDd) in mice, *n* = 6. *p < .05.

We first analyzed TFRC expression in mice heart tissue with pathological cardiac hypertrophy using the recently published single‐cell RNA‐seq data of TAC‐model mouse heart tissue (GSE120064),^20^ and found that five major cellular clusters, including cardiomyocytes (CM), endothelial cells (EC), fibroblasts (FB), macrophages (MP), and smooth muscle cells (SMC) distributed in the HF tissue (Figure 1A). Moreover, we analyzed the TFRC expression in the heart tissue of mice model with pathological cardiac hypertrophy and found that TFRC protein levels in the HF tissues were significantly higher than those in Control, detected by immunofluorescent staining (Figure 1B).

Subsequently, by performing correlation analysis based on TFRC expression using the single‐cell RNA‐seq data of TAC‐model mouse heart tissue (GSE120064),^20^ we found that TFRC expression in CM was significantly and positively correlated to macrophages infiltration in the HF tissues (Figure 1C,D). Furthermore, knockdown of TFRC by tail intravenous injection of AAV‐shR‐TFRC significantly decreased macrophages infiltration which was stained by the macrophage maker F4/80+ in the HF tissues (Figure 1E), and deteriorated heart function with decreased EF% and increased LVEDd measured by echocardiographic evaluation (Figure 1F,G). Collectively, these results indicate that TFRC expression is increased in heart tissue with pathological cardiac hypertrophy and positively associated with macrophage infiltration.

### 3.2 TFRC in cardiomyocytes recruits and activates macrophages by secreting Ccl2 in the process of pathological cardiac hypertrophy

**Figure 2:**
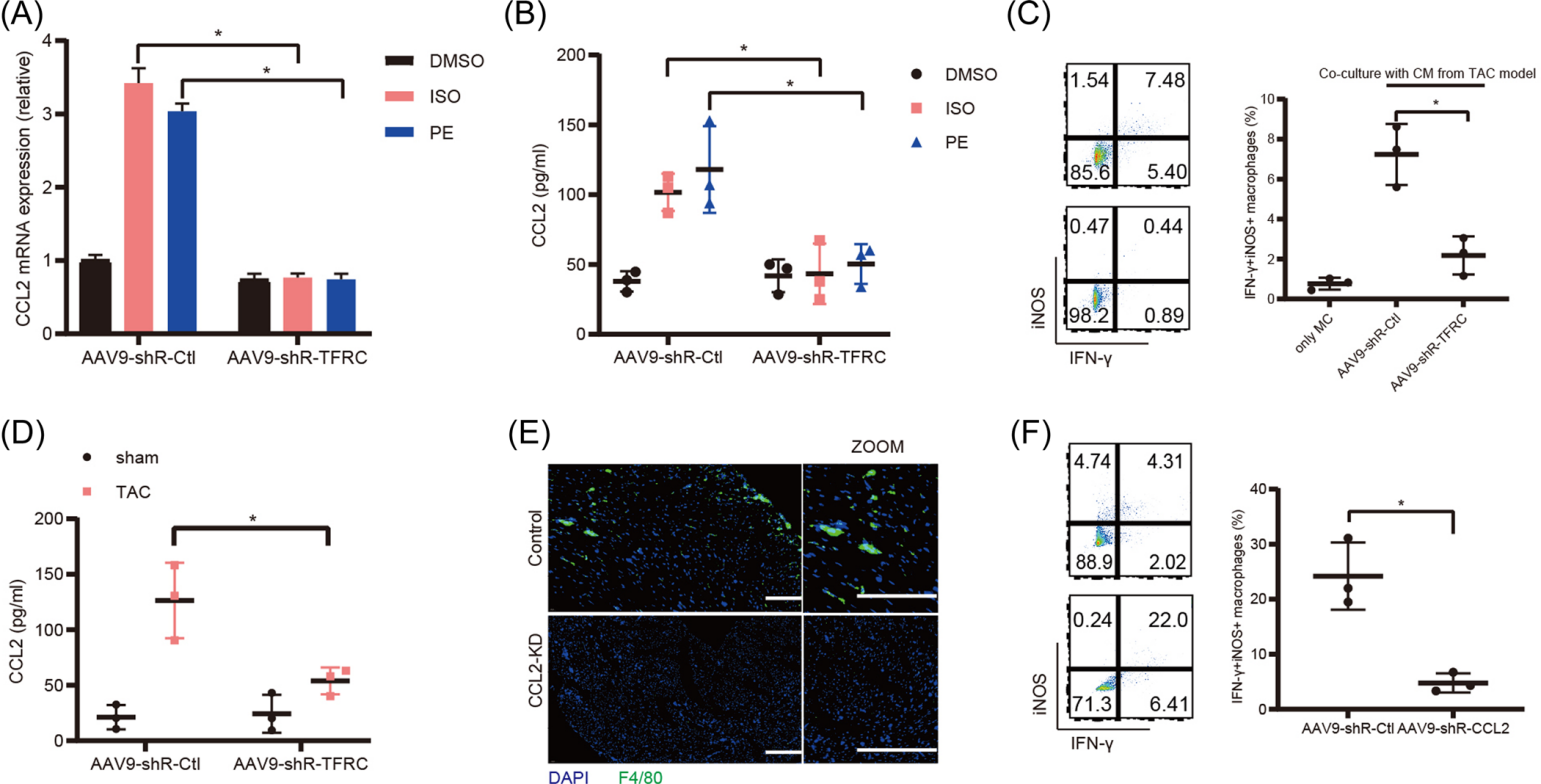
TFRC in cardiomyocytes recruits and activates macrophages by secreting Ccl2 in the process of pathological cardiac hypertrophy. (A) RT‐PCR analysis of Ccl2 mRNA expressions in the primary cardiomyocytes infected with AAV9‐shR‐Ctl or AAV9‐shR‐TFRC for 48 h, and then stimulated with isoproterenol (ISO, 10 μmol/L), phenylephrine (PHE, 50 μmol/L), or DMSO (as control) for 24 h. (B) ELISA analysis of Ccl2 protein expressions in the culture supernatants of the primary cardiomyocytes infected with AAV9‐shR‐Ctl or AAV9‐shR‐TFRC for 48 h, and then stimulated with isoproterenol (ISO, 10 μmol/L), phenylephrine (PHE, 50 μmol/L), or DMSO (as control) for 24 h. (C) Flow cytometry analysis of RAW264.7 macrophages which were co‐cultured with mouse cardiomyocytes which were isolated from the heart tissue of mice injected with AAV9‐shR‐Ctl or AAV‐9‐shR‐TRFC through tail vein for 3 weeks, then subjected to TAC for 4 weeks. (D) ELISA analysis of Ccl2 protein expressions in the serum of mice injected with AAV9‐shR‐Ctl or AAV‐9‐shR‐TRFC through tail vein for 3 weeks, then subjected to TAC for 4 weeks. (E) Immunofluorescence analysis of macrophage distribution stained with anti‐F4/80+ antibodies (Green) in HF tissue of mice injected with AAV9‐shR‐Ctl or AAV‐9‐shR‐Ccl2 through tail vein for 3 weeks, then subjected to TAC for 4 weeks. The bar is 100 μm. (F) Flow cytometry analysis of macrophages isolated from the heart tissue of mice injected with AAV9‐shR‐Ctl or AAV‐9‐shR‐Ccl2 through tail vein for 3 weeks, then subjected to TAC for 4 weeks. *p < .05. CM, cardiomyocyte; MC, macrophage.

Given that cardiomyocytes recruit macrophages upon SARS‐CoV‐2 infection by secreting Ccl2,^32^ we further investigated whether TFRC affecting macrophages distribution in the HF tissue was associated with regulating Ccl2 expression. As shown in Figure 2A,B, Ccl2 mRNA and protein expression levels were significantly increased in the primary cardiomyocytes treated with ISO or PHE to induce myocardial hypertrophy in vitro. Whereas, knockdown of TFRC mediated by AAV9‐ShR‐TFRC evidently blocked the upregulated Ccl2 expression induced by ISO or PHE (Figure 2A,B). To further investigate whether TFRC in cardiomyocytes affected macrophages polarization, cardiomyocytes were isolated from the heart tissue of TAC mice model, and then co‐cultured with the Raw264.7 macrophages for 12 h. Flow cytometry showed that macrophages evidently polarized into pro‐inflammatory M1 subtype in the condition of co‐culture with the primary cardiomyocytes from the HF tissues, detected by M1 maker iNOS antibody and IFN‐γantibody (Figure 2C). However, co‐culture with the cardiomyocytes from the HF tissues of TAC mice injected with AAV9‐shR‐TFRC evidently suppressed macrophages M1 polarization (Figure 2C). Consistently, Ccl2 protein expression was also significantly increased in the serum from the TAC mice model, and knockdown of TFRC by AAV9‐shR‐TFRC significantly decreased Ccl2 expression in the serum (Figure 2D). Moreover, knockdown of Ccl2 by tail intravenous injection of AAV9‐shR‐Ccl2 also significantly decreased macrophages distribution in the HF tissues (Figure 2E). In addition, knockdown of Ccl2 also significantly suppressed macrophages M1 polarization in the HF tissues (Figure 2F). Collectively, these results indicate that TFRC in cardiomyocytes recruits and activates macrophages by secreting Ccl2 in the process of pathological cardiac hypertrophy.

### 3.3 TFRC in cardiomyocytes promotes Ccl2 expression via upregulating STAT3

**Figure 3:**
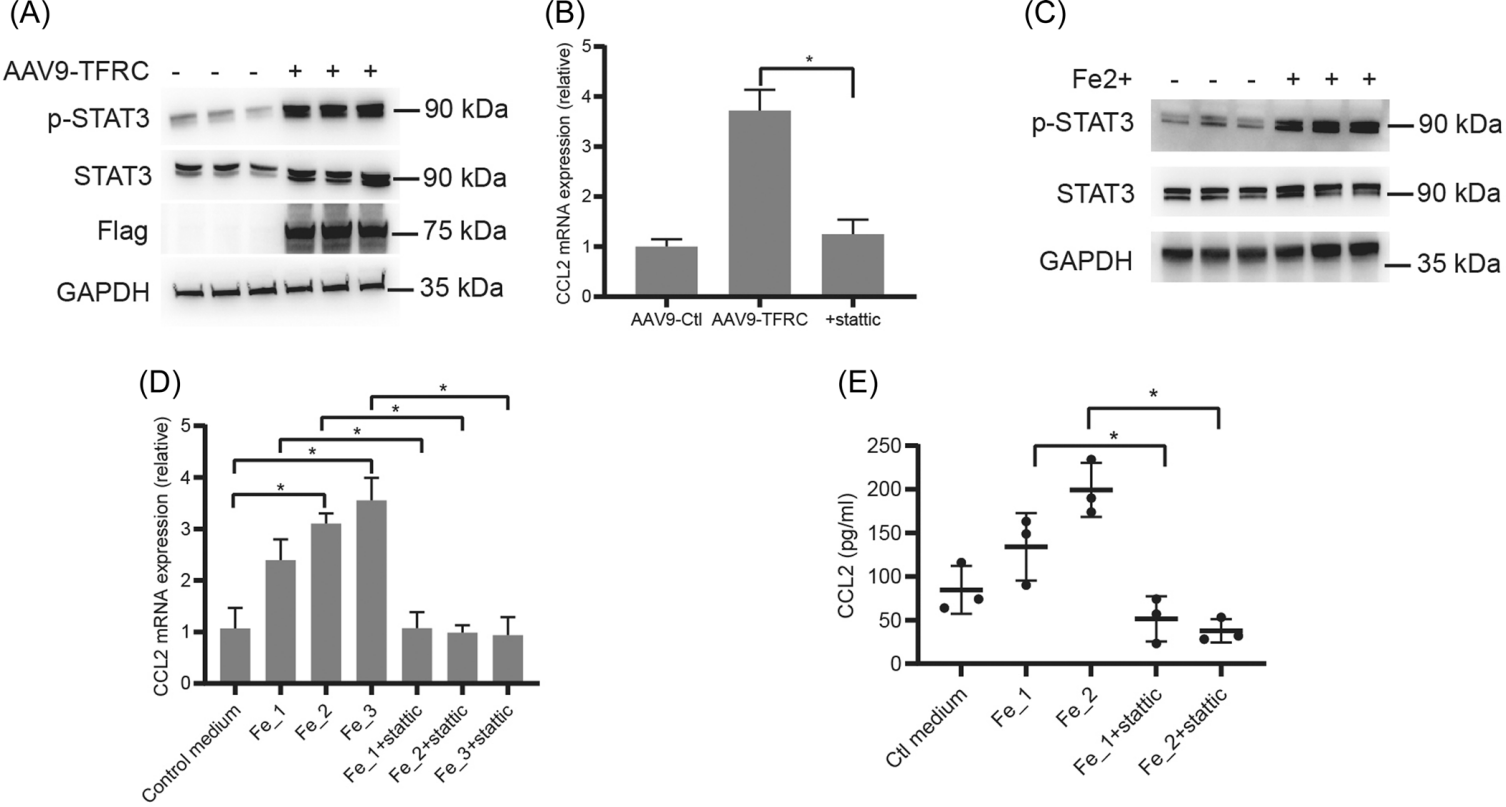
TFRC in cardiomyocytes promotes Ccl2 expression via upregulating STAT3. (A) Western blotting analysis of STAT3 and pSTAT3 protein expressions in the primary cardiomyocytes infected with AAV9‐Flag‐TRFC or AAV9‐Flag‐Ctl for 48 h. (B) RT‐PCR analysis of Ccl2 mRNA expressions in the primary cardiomyocytes infected with AAV9‐shR‐Ctl or AAV9‐shR‐TFRC for 48 h, and then stimulated with Stattic (5 μM) for 2 h. (C) Western blotting analysis of STAT3 and pSTAT3 protein expressions in the primary cardiomyocytes stimulated with 50 μm FeSO_4_ for 4 h. (D) RT‐PCR analysis of Ccl2 mRNA expressions in the primary cardiomyocytes stimulated with FeSO_4_ for 4 h, and then stimulated with Stattic (5 μM) for 2 h. Fe_1, 50 μm FeSO_4_; Fe_2, 75 μm FeSO_4_; Fe_3, 100 μm FeSO_4_. (E) ELISA analysis of Ccl2 protein expressions in the culture supernatants of the primary cardiomyocytes stimulated with FeSO_4_ for 4 h, and then stimulated with Stattic (5 μM) for 2 h. Fe_1, 50 μm FeSO_4_; Fe_2, 75 μm FeSO_4_. *p < .05. TFRC, transferrin receptor.

Previous studies reported that signal transducer and activator of transcription 3 (STAT3) promoted Ccl2 secretion.^33^, ^34^ We then further investigated whether TFRC affected STAT3 expression in cardiomyocytes, and found that overexpression of TFRC in cardiomyocytes by AAV9‐flag‐TFRC infection significantly increased STAT3 and phosphroylated STAT3 (p‐STAT3) expression (Figure 3A), and Ccl2 mRNA expression (Figure 3B). As expected, treatment with the STAT3 inhibitor, stattic, significantly suppressed the promotive effect of TFRC on Ccl2 mRNA expression (Figure 3C). Subsequently, given that TFRC promotes iron uptake through the transferrin cycle,^9^ we further explored whether Fe^2+^ could affect STAT3 and Ccl2 expressions in cardiomyocytes. As expected, Fe^2+^ treatment significantly increased the expressions of STAT3 protein, p‐STAT3 protein, Ccl2 mRNA, and Ccl2 protein in cardiomyocytes (Figure 3C,D,E), and stattic incubation blocked the promotive effects of Fe^2+^ on Ccl2 expressions (Figure 3D,E). Collectively, these results indicate that TFRC in cardiomyocytes promotes Ccl2 expression via upregulating STAT3.

### 3.4 TFRC expression is significantly increased in macrophages during HF and promotes M1 polarization

**Figure 4:**
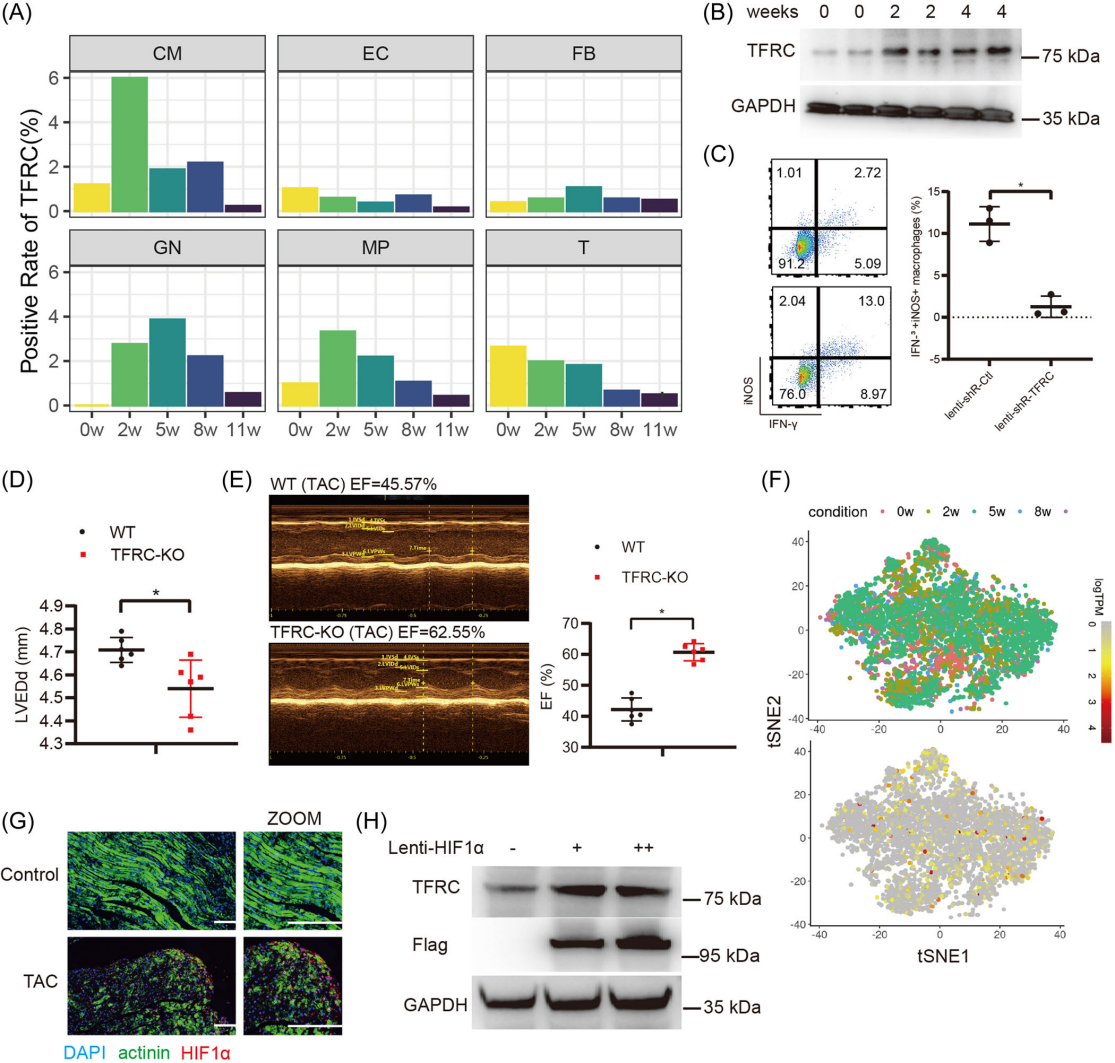
Hypoxia‐inducible factor‐1α (HIF‐1α) promotes TFRC expression in the HF tissue. (A) Analysis of TFRC expression in different cell clusters at different stages of HF. (B) Western blotting analysis of TFRC protein expressions in the mice heart tissue 0/2/4 weeks post‐TAC. (C) Flow cytometry analysis of macrophages isolated from the heart tissue of mice subjected to TAC for 4 weeks, and then infected with Lenti‐shR‐ctl or Lenti‐shR‐TFRC. (D) Detection of LVEDd in mice, *n* = 6. *p < .05. (E) Representative images of echocardiography and quantification of ejection fraction (EF) % (*n* = 6). (F) 2D visualization of HIF‐1α gene expressions at different stages of cardiac hypertrophy. (G) Immunofluorescence analysis of HIF‐1α (Red) expressions in the heart tissue of mice subjected to TAC for 4 weeks. The cardiomyocytes were stained with anti‐α‐actinin antibodies (Green). Nucleus was stained with DAPI (Blue). The bar is 50 μm. (H) Western blotting analysis of TFRC protein expressions in the macrophages isolated from the heart tissue of mice subjected to TAC for 4 weeks and then infected with Letni‐Flag‐HIF‐1α or Lenti‐Flag‐Ctl for 48 h. *p < .05. TAC, transverse aortic constriction; TFRC, transferrin receptor.

Next, we further analyzed the changes of TFRC expression in different cell types at different stages of HF using the published single‐cell RNA‐seq data of TAC‐model mouse heart tissue (GSE120064),^20^ and found that TFRC expressions were significantly increased in the CM (cardiomyocytes), GN (neutrophils), and MP (macrophages) of heart tissue 2 weeks after TAC surgery (Figure 4A). Furthermore, Western blotting analysis confirmed that TFRC expressions were indeed significantly increased in the macrophages isolated from the mice HF tissues post‐TAC (Figure 4B). Moreover, flow cytometry analysis showed that knockdown of TFRC by Lenti‐shR‐TFRC significantly suppressed M1 polarization of macrophages isolated from the mice HF tissues post‐TAC (Figure 4C). In addition, re‐transfusion of macrophages infected with Lenti‐shR‐TFRC by tail intravenous injection significantly improved TAC‐induced heart function injury (indicated by decreased LVEDd and increased EF measured by echocardiographic evaluation), compared to re‐transfusion of macrophages infected with Lenti‐shR‐Ctl (Figure 4D,E). Collectively, these results indicate that TFRC expression is significantly increased in the macrophages during HF and promotes M1 polarization.

### 3.5 Hypoxia‐inducible factor‐1α (HIF‐1α) promotes TFRC expression in the HF tissue

Previous study reported that hypoxia‐inducible factor‐1α (HIF‐1α) promoted TFRC expression in human hepatic cells.^35^ Thus, we investigated whether TFRC expression in the HF tissue was associated with HIF‐1α. As shown in Figure 4F, HIF‐1α expression is induced during HF, analyzed with single‐cell RNA‐seq data of TAC‐model mouse heart tissue (GSE120064).^20^ Furthermore, immunofluorescent staining also confirmed HIF‐1α expression was increased in the HF tissue (Figure 4G). Finally, we found that overexpression of HIF‐1α mediated by Lenti‐flag‐HIF‐1α significantly increased TFRC expression in cardiomyocytes (Figure 4H). Overall, these results indicate that HIF‐1α promotes TFRC expression in the HF tissue.

## 4 DISCUSSION

Cardiac hypertrophy is an initiating link to HF which still seriously endangers human health and is lack of effective therapeutic drugs.^4^, ^5^ In this study, our group first explored the role of TFRC in pathological cardiac hypertrophy, and found that TFRC expression in the HF tissue was significantly increased and positively related to macrophage infiltration in the process of HF, indicating that TFRC plays harmful role in HF. Whereas, recent study reported that TFRC was essential for heart development, and inactivation of TFRC leaded to lethal cardiomyopathy in mice with poor cardiac function, failure of mitochondrial respiration, and ineffective mitophagy,^10^ suggesting that TFRC plays protective roles in lethal cardiomyopathy. In addition, our study found that TFRC in macrophages was required for M1 macrophage polarization which promotes HF progression.^36^, ^37^ Thus, TFRC plays dual roles in the progression of HF. Owing to HF as an complex disease with different pathological mechanisms, clearly clarification of TFRC roles in the different cell clusters at different stages of HF would be essential for developing effective therapeutic drugs for HF.

TFRC is essential for the uptake of iron complexes into cells and for regulating mitophagy.^9^, ^10^ However, iron‐overload in cardiomyocytes is an importantly potential cause of HF.^7^ Besides, TFRC overexpression induces lipid ROS and ferroptosis,^38^, ^39^ which are also important pathological mechanism of HF.^40^ Interestingly, we found that TFRC expression in cardiomyocytes promoted Ccl2 secretion to attract macrophages which worsened heart function. Consistently, cardiomyocytes recruit macrophages upon SARS‐CoV‐2 infection by secreting Ccl2.^32^ Thus, Ccl2 blocking antibody may be a potential therapeutic drug for HF and needs to be further investigated in the following studies. Recently, empagliflozin (EMPA), a selective inhibitor of the sodium glucose co‐transporter 2, which reduced the risk of hospitalization for heart failure and cardiovascular death in type 2 diabetic patients in the EMPA‐REG OUTCOME trial,^41^, ^42^ has been found to improve myocardial strain, and reduces cardiac pro‐inflammatory cytokines via regulating NLRP3 and MyD88‐related pathways in nondiabetic mice treated with doxorubicin.^43^ While, activation of NLRP3 or MyD88‐related pathways induces Ccl2 expression.^44^, ^45^ Whether the cardioprotective effect of EMPA is also related to inhibition of macrophages infiltration should be studied further. In addition, polarized activation affects iron metabolism in macrophages.^46^ M1 macrophages tend to lock iron in the cell and reduce extracellular iron content, while M2 macrophages tend to excrete iron, which contributes to the proliferation of surrounding cells and thus promotes tissue repair.^46^ In this study, we also found that TFRC expression in macrophages were significantly increased in the HF tissue, and were essential for M1 polarization of macrophages. It is indicated that iron metabolism and macrophages polarization are interacted. Although our study clarify the role of TFRC in the development of HF, there are still some questions need to be addressed: (1) Whether TFRC promoting macrophages M1 polarization depends on iron translocation; (2) what is the underlying mechanism of TFRC upregulated expression in macrophages in the progression of HF? (3) Whether specific knockdown of TFRC in macrophages may be feasible for HF therapy. In our future studies, macrophages‐specific TFRC knockout mice should be constructed to answer these questions.

## 5 CONCLUSION

Our group investigated the role of TFRC in the development of HF, and found TFRC in cardiomyocytes promoted HF development through inducing macrophage infiltration and activation via the STAT3‐Ccl2 signaling, and TFRC expression in cardiomyocytes was regulated by HIF‐1α during HF. However, inactivation of TFRC leaded to lethal cardiomyopathy in mice. Thus, using TFRC inhibitor alone may be not helpful for regressing HF.

## AUTHOR CONTRIBUTIONS

Pan Yanyun, Yang Jinxiu, Dai Jin, Xu Xiaoming, Zhou Xinbin, and Mao Wei conceptualized and realized the study. Pan Yanyun, Yang Jinxiu, Dai Jin, Xu Xiaoming, Zhou Xinbin, and Mao Wei involved in acquisition and analysis of data and drafted the manuscript. Pan Yanyun, Zhou Xinbin, and Mao Wei revised the manuscript and obtained fund.

## CONFLICTS OF INTEREST STATEMENT

The authors declare no conflict of interest.

## ETHICS STATEMENT

All animal experiments (No. 20220328‐17) were approved by the institutional animal care committee of the First Affiliated Hospital of Zhejiang Chinese Medical University and complied with the Guide for the Care and Use of Laboratory Animals.

## Data Availability

The data used to support this study are included within the article.
