# Correction to “TFRC in Cardiomyocytes Promotes Macrophage Infiltration and Activation During the Process of Heart Failure Through Regulating Ccl2 Expression Mediated by Hypoxia Inducible Factor‐1Α”

**DOI:** 10.1002/iid3.70262

**Published:** 2025-09-11

Pan Y, Yang J, Dai J, Xu X, Zhou X, Mao W. TFRC in cardiomyocytes promotes macrophage infiltration and activation during the process of heart failure through regulating Ccl2 expression mediated by hypoxia inducible factor‐1α. Immunity, Inflammation and Disease 2023; 11:e835. doi:10.1002/iid3.835

In the “Figure Legend” section of Figure 1B, the text “The bar is 50 μm.” was incorrect. This should have read: “The bar of the left‐hand panel is 50 μm. The right‐hand panel depicts a magnified view of selected regions from the left‐hand images, with the bar of 100 μm.”. In the “Figure Legend” section of Figure 1C, the annotation of Crosses was inadvertently omitted. This should have read: (C) The correlation analysis of different cells in HF tissue. The right‐side vertical bar chart employs a gradient color scale (red to blue) to quantify correlation strength, with red indicating positive and blue negative correlations. The y‐axis spans from −1 to 1, where the single bar reaching maximum amplitude (value = 1) demonstrates the strongest positive correlation. The visual hierarchy is further clarified by: (1) Circular markers whose size corresponds to absolute correlation magnitude; (2) Cross symbols denoting statistical insignificance. In the “Figure Legend” section of Figure 1D, the annotation of the x‐axis was inadvertently omitted. This should have read: (D) The correlation analysis of TFRC expression and different cells in HF tissue (CM, cardiomyocytes; EC, endothelial cells; FB, fibroblasts; MP, macrophages; GN, neutrophile granulocyte; T, T cells). The x‐axis represents the relative mRNA expression level of TFRC, while the y‐axis illustrates the corresponding cell population proportions. The authors also admitted to an image compilation error in the subpanels of Figure 1B and were able to provide the original images. The authors confirm that all the experimental results and corresponding conclusions mentioned in the paper remain unaffected. The corrected Figure 1 is shown as follows.

**Figure 1:**
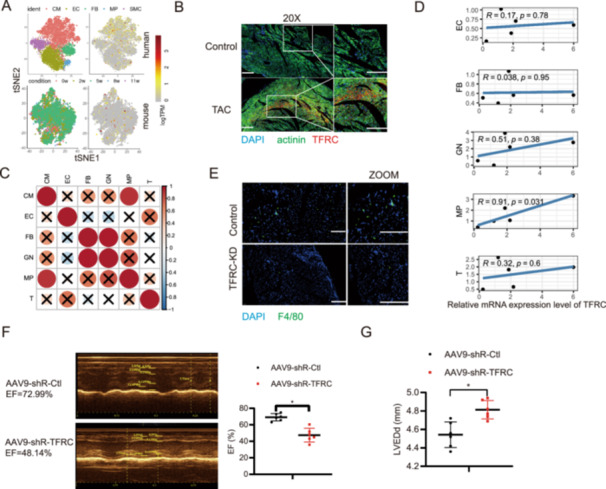
.

Corrected Figure 1

The authors apologize for these errors.

